# Recombinant human interleukin-7 reverses T cell exhaustion ex vivo in critically ill COVID-19 patients

**DOI:** 10.1186/s13613-022-00982-1

**Published:** 2022-03-05

**Authors:** Frank Bidar, Sarah Hamada, Morgane Gossez, Remy Coudereau, Jonathan Lopez, Marie-Angelique Cazalis, Claire Tardiveau, Karen Brengel-Pesce, Marine Mommert, Marielle Buisson, Filippo Conti, Thomas Rimmelé, Anne-Claire Lukaszewicz, Laurent Argaud, Martin Cour, Guillaume Monneret, Fabienne Venet, RICO Study Group, Remi Pescarmona, Lorna Garnier, Christine Lombard, Magali Perret, Marine Villard, Sébastien Viel, Valérie Cheynet, Elisabeth Cerrato, Estelle Peronnet, Jean-François Llitjos, Laetitia Itah, Inesse Boussaha, Françoise Poitevin-Later, Christophe Malcus, Marine Godignon, Florent Wallet, Marie-Charlotte Delignette, Frederic Dailler, Marie Simon, Auguste Dargent, Pierre-Jean Bertrand, Neven Stevic, Marion Provent, Laurie Bignet, Valérie Cerro, Jean-Christophe Richard, Laurent Bitker, Mehdi Mezidi, Loredana Baboi

**Affiliations:** 1 Joint Research Unit HCL-bioMérieux, EA 7426 “Pathophysiology of Injury-Induced Immunosuppression”, Université Claude Bernard Lyon, 1-Hospices Civils de Lyon-bioMérieux, 69003 Lyon, France; 2 Anesthesia and Critical Care Medicine Department, Edouard Herriot Hospital, Hospices Civils de Lyon, 69437 Lyon, France; 3 Immunology Laboratory, Hôpital E. Herriot-Hospices Civils de Lyon, 5 place d’Arsonval, 69437 Lyon Cedex 03, France; 4 Centre International de Recherche en Infectiologie (CIRI), Inserm U1111, CNRS, UMR5308, Ecole Normale Supérieure de Lyon, Université Claude, Bernard-Lyon 1, Lyon, France; 5 Biochemistry and Molecular Biology Laboratory, Lyon-Sud University Hospital-Hospices Civils de Lyon, Chemin du Grand Revoyet, Pierre-Benite, France; 6 Centre d’Investigation Clinique de Lyon (CIC 1407 Inserm), Hospices Civils de Lyon, 69677 Lyon, France; 7 Medical Intensive Care Department, Hospices Civils de Lyon, Edouard Herriot Hospital, 69437 Lyon, France

**Keywords:** T lymphocytes, Exhaustion, SARS-CoV-2, Critically ill, Interleukin-7, Immunostimulation

## Abstract

**Background:**

Lymphopenia is a hallmark of severe coronavirus disease 19 (COVID-19). Similar alterations have been described in bacterial sepsis and therapeutic strategies targeting T cell function such as recombinant human interleukin 7 (rhIL-7) have been proposed in this clinical context. As COVID-19 is a viral sepsis, the objectives of this study were to characterize T lymphocyte response over time in severe COVID-19 patients and to assess the effect of ex vivo administration of rhIL-7.

**Results:**

Peripheral blood mononuclear cells from COVID-19 patients hospitalized in intensive care unit (ICU) were collected at admission and after 20 days. Transcriptomic profile was evaluated through NanoString technology. Inhibitory immune checkpoints expressions were determined by flow cytometry. T lymphocyte proliferation and IFN-γ production were evaluated after ex vivo stimulation in the presence or not of rhIL-7. COVID-19 ICU patients were markedly lymphopenic at admission. Mononuclear cells presented with inhibited transcriptomic profile prevalently with impaired T cell activation pathways. CD4 + and CD8 + T cells presented with over-expression of co-inhibitory molecules PD-1, PD-L1, CTLA-4 and TIM-3. CD4 + and CD8 + T cell proliferation and IFN-γ production were markedly altered in samples collected at ICU admission. These alterations, characteristic of a T cell exhaustion state, were more pronounced at ICU admission and alleviated over time. Treatment with rhIL-7 ex vivo significantly improved both T cell proliferation and IFN-γ production in cells from COVID-19 patients.

**Conclusions:**

Severe COVID-19 patients present with features of profound T cell exhaustion upon ICU admission which can be reversed ex vivo by rhIL-7. These results reinforce our understanding of severe COVID-19 pathophysiology and opens novel therapeutic avenues to treat such critically ill patients based of immunomodulation approaches. Defining the appropriate timing for initiating such immune-adjuvant therapy in clinical setting and the pertinent markers for a careful selection of patients are now warranted to confirm the ex vivo results described so far.

*Trial registration* ClinicalTrials.gov identifier: NCT04392401 Registered 18 May 2020, http:// clinicaltrials.gov/ct2/show/NCT04392401.

**Supplementary Information:**

The online version contains supplementary material available at 10.1186/s13613-022-00982-1.

## Introduction

The Coronavirus disease 2019 (COVID-19) pandemic caused by the highly infectious severe acute respiratory syndrome coronavirus 2 (SARS-CoV-2) has now challenged public health for almost two years. The most severe forms evolve towards acute respiratory distress syndrome (ARDS) requiring care in intensive care unit (ICU). A better understanding of the immune response in these critically ill patients is of major importance to guide therapeutics as a high mortality is still reported in this population [1] and prolonged ICU lengths of stay ultimately lead to ICU saturation in time of pandemic [2].

A fully competent immune system is mandatory to fight against infections. Evidence now suggest that severe forms of COVID-19 are associated with the development of immune alterations. For example, impaired and delayed type I and type III interferon (IFN) responses have been described in severe COVID-19 patients [3]. In addition, critically ill COVID-19 patients display features of compromised adaptive immunity mainly characterized by profound lymphopenia; which depth and persistence associate with poor prognosis [4–6].

Major lymphocyte alterations have been described in other clinical contexts such as bacterial sepsis, chronic viral infections and cancer [7]. In addition to marked lymphopenia, these patients exhibit severely altered T cell effector functions with decreased proliferation and cytokine production ex vivo and sustained expression of inhibitory receptors such as programmed cell death protein (PD-1) or programmed death-ligand 1 (PD-L1) [8]. These alterations of T lymphocyte response have been characterized as T cell exhaustion [9, 10]. Similar features of T cell exhaustion have been described in patients after severe SARS-CoV-2 infections [11]. However their onset of appearance and their persistence over ICU stay remain unclear.

In patients with bacterial sepsis, immunotherapies targeting T cell exhaustion have been suggested to improve T cell functions and patients’ prognostic [7]. Among these, interleukin 7 (IL-7) is a pleiotropic cytokine involved in T cell survival and expansion [12]. Several in vivo and ex vivo studies reported a beneficial effect of recombinant interleukin 7 (rhIL-7) in sepsis through restoration of lymphocyte proliferation, IFN-γ production [13–15] or metabolic reprogramming of exhausted CD4 + T cells [16]. So far only few studies investigated rhIL-7 beneficial effect on T cell alterations in COVID-19 patients. One study showed that IL-7 administered ex vivo restored T cell IFN-ɣ production [11] and compassionate use of this treatment was reported in two studies [17, 18].

In this context, the objectives of the present work were (i) to characterize T cell transcriptome, phenotype and functions over time in a cohort of critically ill COVID-19 patients and (ii) to evaluate ex vivo the capacity of rhIL-7 therapy to improve T cell functions in critically ill COVID-19 patients.

## Materials and methods

### Sampling and cell isolation

COVID-19 patients belong to the RICO (REA-IMMUNO-COVID) study registered at NCT04392401 and approved by local Institutional Review Board for ethics (“Comité de Protection des personnes d’Ile-de-France”) [19]. All patients were prospectively included. The study group consisted in consecutive ICU-hospitalized patients in seven tertiary hospitals in France. Inclusion criteria were: (1) patients aged > 18 years, (2) hospitalization in ICU for SARS-CoV-2 pneumopathy, (3) first hospitalization in ICU for COVID-19, (4) positive diagnosis of SARS-CoV-2 infection carried out by PCR or by another approved method in at least one respiratory sample, (5) sampling in the first 48 h after admission to ICU feasible and (6) patient or next of kin informed of the terms of the study and has not objected to participating. Blood samples were collected within the first 48 h after ICU admission (Day 0: D0) and between day 20 and day 25 (D20) in EDTA-anticoagulated blood bottles. Reference values were obtained from a cohort of 15 healthy volunteers (HV) after informed consent was given. Healthy volunteers’ (10 women, 5 men) mean age was 38 years. Peripheral blood mononuclear cells (PBMCs) from patients and donors were isolated by Ficoll-Paque PREMIUM (GE Healthcare) density gradient centrifugation and cryopreserved in a − 80 °C freezer.

### Reagents

Anti–CD2-CD3-CD28 coated beads (T cell activation/expansion kit human) were purchased from Miltenyi Biotec (Gladbach, Germany). Alexa Fluor 750 or Fluorescein isothiocyanate-conjugated anti-CD3, Allophycocyanin or Pacific Blue–conjugated anti-CD4, Krome orange-conjugated anti-CD8, Phycoerythrin-conjugated anti-CD16/56, Pacific Blue-conjugated anti-CD19, Krome Orange-conjugated anti-CD45, Allophycocyanin-conjugated anti-CD14, Phycoerythrin-Cyanin-7-conjugated anti-PD-L1, Phycoerythrin-conjugated anti-CTLA-4 (cytotoxic T-lymphocyte-associated protein 4), were purchased from Beckman Coulter (Hialey, FL). Allophycocyanin-conjugated anti-PD1 and Phycoerythrin-Dazzle 594 conjugated anti-TIM-3 (T-cell immunoglobulin and mucin containing protein-3) antibodies were purchased from Biolegend (San Diego, CA). rhIL-7 was purchased from R&D Systems (Minneapolis, MN). RNAse-free Dnase set was purchased from Qiagen (Hilden, Germany). Click-it kit Ethynyl-2′-deoxyuridine (EdU) AF88 was purchased from ThermoFisher (Whaltham, MA).

### Thawing and phenotyping of PBMCs

Cryovials containing PBMCs were thawed in water bath and quickly resuspended in RPMI medium with RNAse-free DNAse prewarmed at 37 °C. Cell number and viability were assessed via flow cytometry through propidium iodide exclusion technique. Median viability calculated on all samples after cell thawing was 80%. Percentages of B cells, NK cells, T cells and monocytes were determined by flow cytometry. PD-1, PD-L1, CTLA-4 and TIM-3 expressions were analyzed on CD3 + CD4 + and CD3 + CD8 + cells. Results were expressed as percentages of cells expressing each marker. Samples were run on a Navios flow cytometer (Beckman Coulter). Calibration beads (Flow Check and Flow Set, Beckman Coulter) were run daily to check for routine alignment, day-to-day stability, and long-term performance validation.

### Gene expression analysis

Total mRNA was extracted from 2.10^6^ PBMCs using Quick-RNA™ Microprep Kit (Zymo research) according to manufacturer’s instructions. We analyzed 200 ng of total RNA from each sample using the nCounter® PanCancer Immune Profiling Panel (NanoString) according to manufacturer’s instructions.

Raw data were prepared for gene expression analysis as described elsewhere [20]. For each sample, eight negative probes and six serial concentrations of positive control probes were included in the panel and used in the preparation of raw data. Briefly, a first step of normalization for variation associated with the technical platform (batch effect) was performed using the internal positive controls. For each sample the geometric mean of internal positive controls was calculated. We then determined a scaling factor per sample as the ratio of the average across all geometric means and the geometric mean of the sample. For each sample, all gene counts were then multiplied by the corresponding scaling factor. Next, for each sample we calculated the background level as the median + 2 standard deviations across the eight negative probe counts. For each sample, background level was subtracted to each gene.

Then, normalization for differences in RNA input used the same method as in the positive control normalization, but geometric means were calculated over three housekeeping genes (EIF2B4, HDAC3, TBP) selected from the forty candidate reference genes provided by NanoString. The number of housekeeping genes to be included was determined using geNorm method [21] and the three most relevant genes were selected using NormFinder method [22].

Finally, negative expression values were converted to zero, and genes for which more than 50% of samples presented with a null value were excluded from the analysis (i.e. 39 genes).

### T cell proliferation assay and IFN-γ production

The number of PBMCs per well was adjusted to 1.10^6^ cells/ml resuspended in complete culture media (RPMI 1640 medium with HEPES, L-glutamine, 10% human AB plasma, 20 mg/ml streptomycin and 2.5 mg/ml fungizone). Cells were stimulated with anti–CD2-CD3-CD28 Abs coated beads (ratio beads/cells = 2:1) and/or with rhIL-7 (100 ng/ml) for 72 h at 37 °C, 5% CO_2_. All samples were performed in duplicates. EdU click-it reaction was performed using EdU Click-it kit (Thermo Fisher) according to the manufacturer’s instructions [23]. CD3 + , CD4 + and CD8 + T cell proliferation was analyzed by monitoring EdU-AF488 incorporation into cells. Results were expressed as percentages of cells incorporating EdU.

IFN-γ production was measured in corresponding cell culture supernatants via standardized Enzyme Linked Fluorescent Assay (VIDAS, bioMérieux). Results were expressed as international units per mL (IU/mL).

### Statistical analysis, data visualization and software

Continuous variables were expressed as medians and interquartile ranges (IQR). Comparisons between results in patients and HV were made using non parametric Mann–Whitney U tests. Wilcoxon signed-rank test was used for paired comparisons between results obtained at D0 and D20 in patients or to assess effect of rhIL-7 within a group.

Differentially expressed genes were visualized on volcano plots and statistical differences were selected based on Log2 Fold Change (i.e. ≤ -−2 or ≥  + 2) and –Log 10 P value (≤ 1.3). Number of significantly downregulated and upregulated genes were represented on Venn diagrams. Ingenuity Pathways Analysis tool (IPA, Ingenuity® Systems, https://analysis.ingenuity.com) was applied on the list of differentially expressed genes to assess canonical pathways. This analysis was performed using both the HTA and U133 gene repertoires. The significant pathways were extracted from the HTA gene repertoire because the results were similar but more complete and informative than the U133 repertoire.

Except for IPA tool, all statistical analyses were performed using R 4.0.4 (The R foundation for Statistical Computing, Vienna, Austria). A *p*-value < 0.05 was considered statistically significant. All tests realized were two-sided.

## Results

### Patients’ characteristics

Twenty patients were included in the study with a median age of 71 [IQR: 66, 72] years. They were admitted in ICU 9 days [IQR: 7, 11] after symptoms onset. Clinical characteristics are presented in Table 1. Overall, they presented with severe forms of COVID-19 pneumonia as 90% required invasive mechanical ventilation during their ICU stay and 28-day mortality was 35%. At D20, all selected patients were still hospitalized in ICU and 70% remained under mechanical ventilation. At this time point, SOFA score was 6.5 [5–8].

**Table 1:** Clinical and biological characteristics of COVID-19 patients

	All patients (*n* = 20)		
Demographics			
Gender, male (%)	14 (70.0)		
Age	71 [66, 72]		
Body mass index	32 [28–36]		
Comorbidities			
Comorbidity (%)	9 (50.0)		
Immunosuppressive therapy (%)	1 (5.6)		
Diabetes (%)	4 (23.5)		
Delay from symptom onset, days	9.0 [7.0, 11.0]		
Severity scores at admission			
SOFA	5.0 [4.0, 7.0]		
SAPS II	37.0 [30.3, 44.3]		
PaO2/FiO2	116.0 [68.3, 151.5]		
Organ failures during ICU stay			
Invasive mechanical ventilation (%)	18 (90)		
Vasoactive drugs (%)	15 (83.3)		
Renal replacement therapy (%)	4 (22.2)		
Follow-up			
Concurrent bacterial infection at D0 (%)	9 (45)		
ICU-acquired infection before D20 (%)	15 (75)		
SOFA score at D20	6.5 [5.00, 8.00]		
Corticotherapy in ICU (%)	18 (90)		
Mechanical ventilation duration	21.5 [16.5, 32.5]		
Days in ICU	28.0 [24.3, 39.5]		
Days in Hospital	32.5 [25.8, 45.8]		
Day-28 mortality	7 (35)		
Day-90 mortality	9 (45)		
Status at time of sampling	D0	D20	
Invasive mechanical ventilation	8 (40)	14 (70)	
Hospitalized in ICU	20 (100)	20 (100)	
Laboratory values	D0 (*n* = 19)	D20 (*n* = 18)	p-value
Lymphocyte (cells/µL)	534 [392, 751]	1 055 [713, 1 488]	0.003
CD3 + lymphocytes (cells/µL)	355 [214, 445]	760 [422, 967]	0.001
CD4 + lymphocytes (cells/µL)	189 [117, 316]	415 [228, 590]	0.003
CD8 + lymphocytes (cells/µL)	103 [82, 198]	262 [114, 430]	0.018
mHLA-DR expression (Ab/C)	6 548 [4 622, 11 704]	6 855 [5 946, 13 897]	0.741

The results are shown as medians and interquartile ranges [Q1–Q3] for continuous variables or numbers and percentages for categorical variables. SOFA: Sepsis-related organ failure assessment. SAPS II: Simplified acute physiology score II. ICU: Intensive care unit. Continuous laboratory variables were compared with paired Mann–Whitney *U* test and *p*-value reported is two-sided. Values were missing for 2 patients at D0 and one patient at D20. Normal values obtained from routine Immunology Lab for total lymphocyte count: [1000–2800] cells/µL, for T lymphocytes: [595 –1861] cells/µL, for CD4 + T cells: [336–1 126] cells/µL, for CD8 + T cells: [125–780] cells/µL, for mHLA-DR: [13,500–45,000] Ab/C (number of antibodies-bound per cell)

Lymphocyte absolute count was notably decreased in COVID-19 patients at D0 (534 cells/μL [IQR: 392–751]) compared to normal laboratory values (1 000–2 800 cells/μL). It significantly increased over time to ultimately normalize at D20. COVID-19-induced lymphopenia affected all T lymphocyte subsets as both CD4 + and CD8 + T cell numbers were markedly decreased in patients at D0. CD4 + and CD8 + T cell counts also significantly increased over time to normalize at D20. Besides, mHLA-DR expression was markedly decreased in COVID-19 patients (Table 1). Overall this suggests the presence of an immunosuppressive profile in critically ill patients after SARS-Co-V2 infection.

### Transcriptomic profile of mononuclear cells in severe COVID-19 patients

**Fig. 1:**
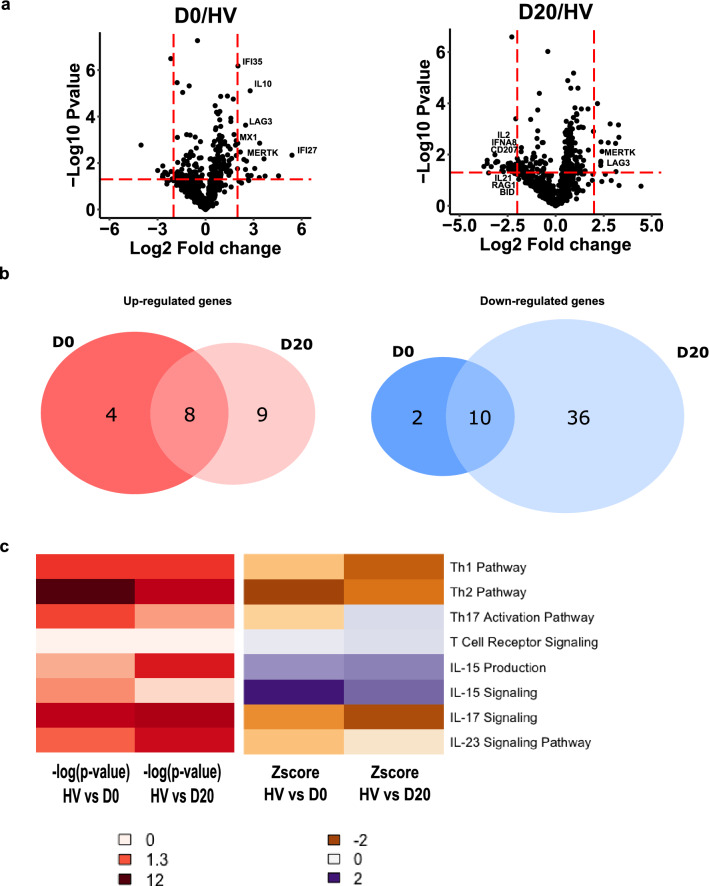
Transcriptomic profile of mononuclear cells in severe COVID-19 patients. RNA extracted from PBMCs of COVID-19 patients at day 0 and day 20 (*n* = 10) and healthy volunteers (HV, *n* = 10) was analyzed through NanoString technology. **a** Volcano plots of differentially expressed genes between patients sampled at D0 or at D20 and HV are showed. Limits of significance are illustrated by red dotted lines (i.e. Log2 Fold Change = −2 or + 2 and −Log 10 *P* value = 1.3). Selected genes are mentioned. **b** Venn diagrams of significantly up-regulated (left diagram, *n* = 21) or down-regulated (right diagram, *n* = 48) genes between patients and HV are showed. **c** Ingenuity Pathway Analysis was applied on the list of differentially expressed genes at D0 and D20. Heatmaps of Log 10 *P*-value (from white indicating the absence of significance to dark red indicating a strong significance) and *Z*-score (from orange indicating a down-regulation to purple indicating an up-regulation) for pathways related to T cell activation at D0 and D20 are presented

We first compared gene expression profiles of PBMCs between healthy volunteers and COVID-19 patients at ICU admission (D0) and D20. Volcano plots unveiled transcriptional differences between COVID-19 patients and HV at both time-points (Fig. 1a). Among the panel of genes analyzed with NanoString in critically ill COVID-19 patients, the number of downregulated genes (*n* = 48) was largely superior to the number of upregulated mRNA (*n* = 21, Fig. 1b). The complete lists of significantly up and down regulated genes are reported in Additional file 1: Tables S1 and S2. We identified differential expressions of genes associated with immunoregulation and impaired adaptive immune response. For instance, mRNA levels of immunoregulatory molecules such as *LAG3, MERTK* or *IL-10* were upregulated while mRNA levels of molecules related to T cell effector functions such as *IL-2*, *IL-21, IL13RA2* or *RAG-1* were downregulated in patients compared with controls. In line, Ingenuity Pathway Analysis showed that pathways related to T cell activation (notably Th1, Th2 and Th17 pathways) were inhibited in patients compared to HV (Fig. 1c). The list of pathways differentially expressed at D0 and D20 compared to HV are reported in Additional file 1: Table S3.

Altogether, these data show the inhibited transcriptomic profile of mononuclear cells in COVID-19 patients. They suggest that T cell function may be impaired after severe SARS-Co-V2 infection.

### Immune checkpoint expressions on T cells in COVID-19 patients

**Fig. 2:**
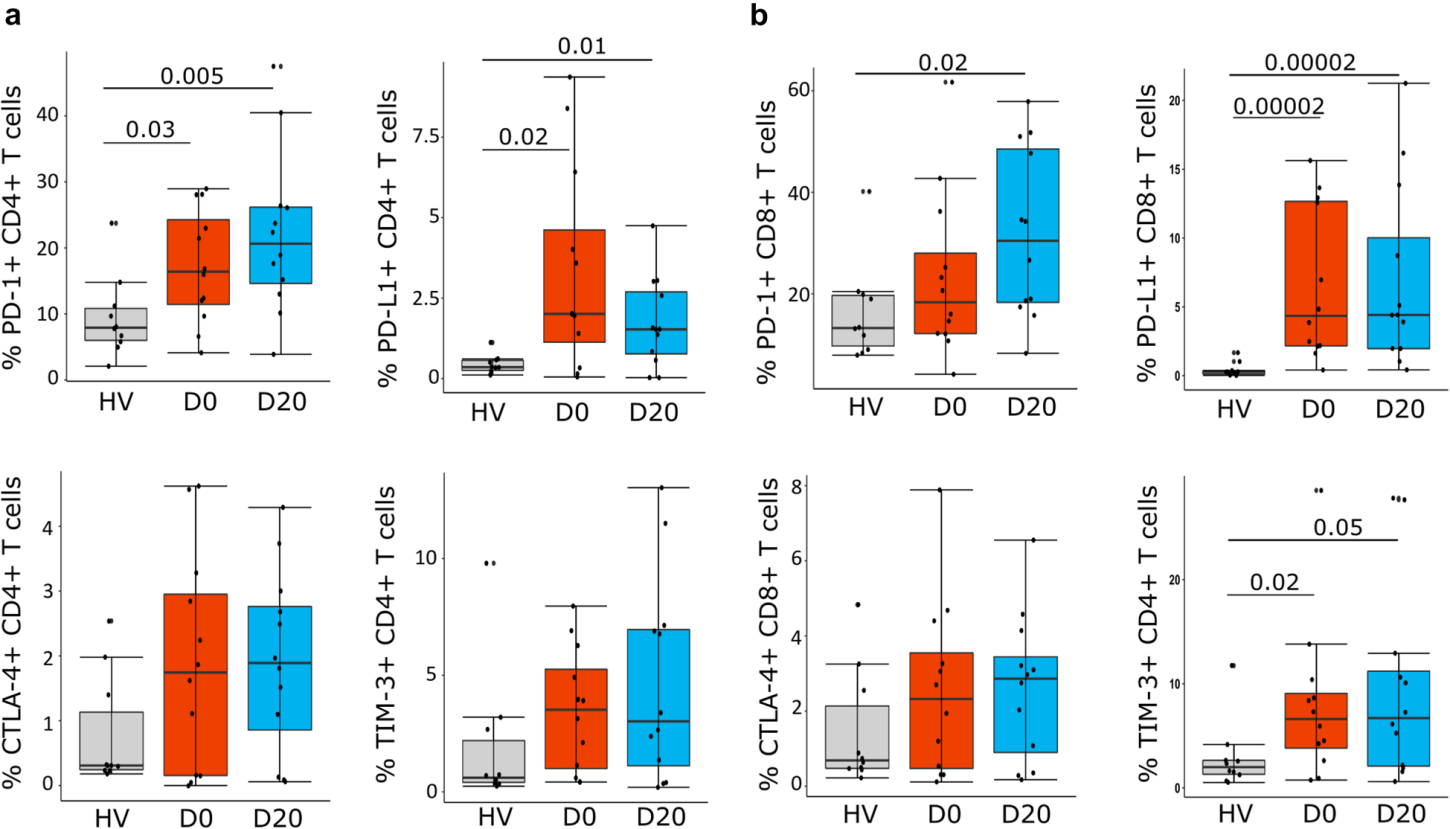
Immune-inhibitory receptors expressions on CD4 + and CD8 + T cells in severe COVID-19 patients. PD-1, PD-L1, CTLA-4 and TIM-3 expressions were assessed by flow cytometry in PBMCs from COVID-19 patients (*n* = 12) at day 0 (D0) and day 20 (D20) and healthy volunteers (HV, *n* = 10). Results are presented as individual values and boxplots. **a** Expressions on CD4 + T cells are presented. **b** Expressions on CD8 + T cells are presented. Nonparametric Mann–Whitney *U* test was used to compare results between HV and COVID-19 patients. Only statistically significant differences are shown

We next evaluated the expressions of the exhaustion markers PD-1, PD-L1, CTLA-4 and TIM-3 on CD4 + and CD8 + T cells in COVID-19 patients sampled at D0 and D20 compared to HV. Flow cytometry analyses showed that the percentages of PD-1 + and PD-L1 + cells were significantly increased among CD4 + T cells at D0 and D20 (Fig. 2a). Similarly, CD8 + T cells displayed an exhaustion phenotype as they presented sustained expressions of PD-1 and PD-L1 (Fig. 2b). CTLA-4 expression was slightly increased in patients versus controls, but the difference did not reach statistical significance. Finally, TIM-3 expression was significantly increased on CD8 + T cells at ICU admission and persisted over ICU course (Fig. 2b).

To note, we found a strong negative correlation between PD-1 expression on CD4 + T cells and the absolute CD4 + T cells count at D0 (Spearman’s correlation *ρ* =−0.83, *p* = 0.0009) and at D20 (*ρ * = −0.62, *p* = 0.028).

Thus CD4 + and CD8 + T cells in severe COVID-19 patients presented with concomitant over-expressions of co-inhibitory molecules PD-1, PD-L1, CTLA-4 and TIM-3 in accordance with the development of a state of T exhaustion after severe SARS-Co-V2 infection.

### T cell proliferation and IFN-γ production in COVID-19 patients

Next we assessed T cell function (i.e. proliferation and IFN-γ production after TCR stimulation ex vivo) in COVID-19 patients sampled at D0 and D20 compared to HV.

**Fig. 3:**
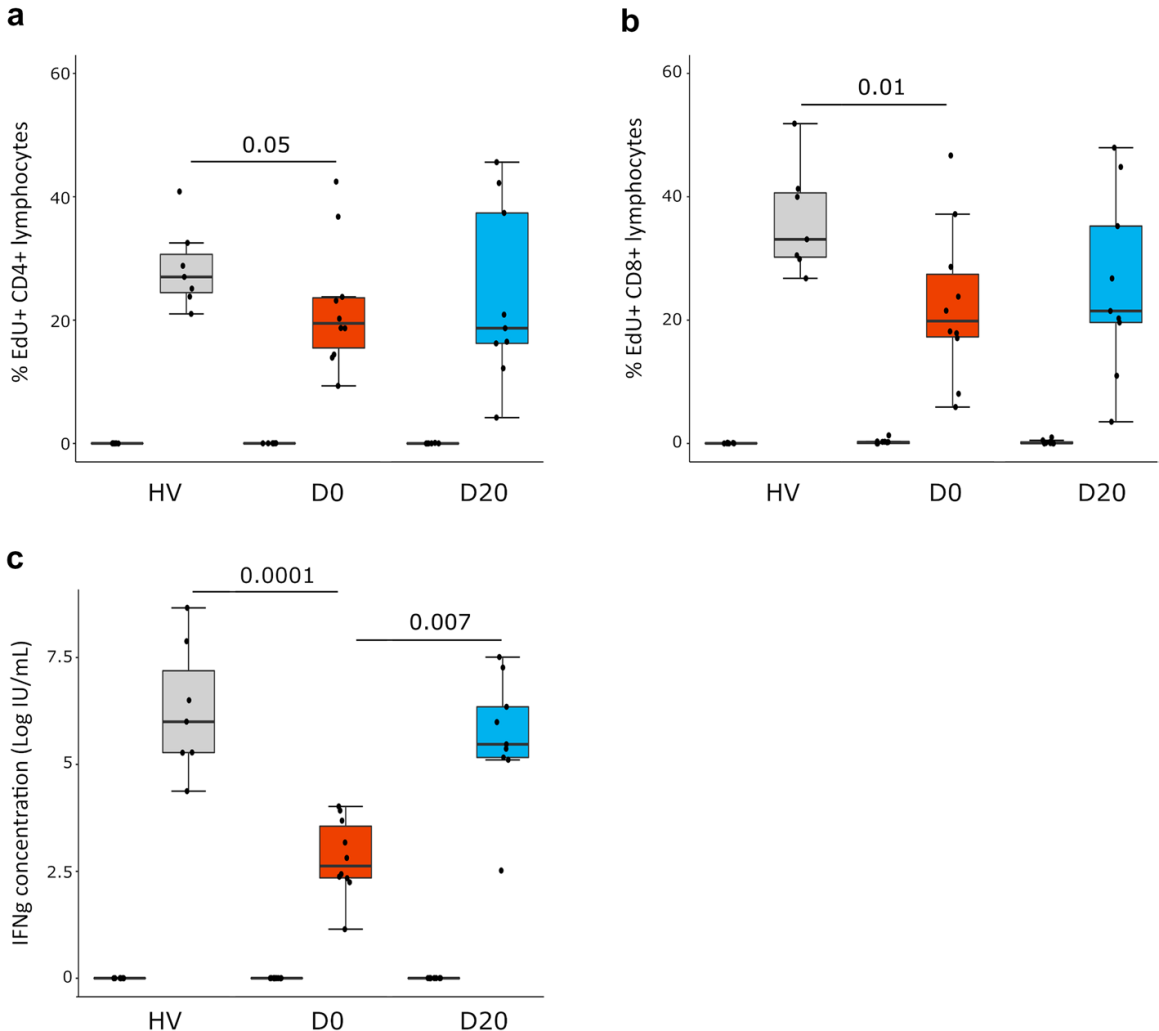
Lymphocyte proliferation and IFN-γ production after TCR stimulation in severe COVID-19 patients. PBMCs were collected from healthy volunteers (HV, *n* = 7) and COVID-19 patients at D0 and D20 (*n* = 10). Cells were cultured for 3 days in the presence (TCR stimulation) or not (Control) of anti-CD2/CD3/CD28 Ab-coated beads (ratio of beads/cells = 2:1). Cellular proliferation was assessed by monitoring EdU AF488 incorporation into cells and expressed as percentages of cells incorporating EdU. IFN-γ was measured in cell culture supernatants after stimulation. Results are presented as individual values and boxplots. **a** CD4 + T cell proliferation is presented. **b** CD8 + T cell proliferation is presented.** c** Natural log transformed IFN-γ production in culture supernatants is presented. Nonparametric Mann–Whitney *U* test was used to compare results between HV and patients. Wilcoxon signed-rank test was used for paired comparisons between D0 and D20. Only statistically significant differences are shown

Both CD4 + and CD8 + T cell proliferations were significantly decreased at ICU admission in patients compared to HV (i.e. 19.5% [IQR: 15.5–23.6]) *vs* 27% [IQR: 24.5–28.5], *p* < 0.05 for CD4 + T cells and 19.9% [IQR: 17.3–27.4] *vs* 33.1% [IQR: 30.2–40.6], *p* < 0.05 for CD8 + T cells). Seven out of 10 patients presented with T cell proliferation responses below the lowest value of HV (Fig. 3a and b). In contrary, at D20, while median T cell proliferation tended to remain lower in patients compared to HV; this difference did not reach statistical significance. To note; the proliferative response was not correlated with absolute lymphocyte count of both CD4 + and CD8 + T cells (Spearman’s correlation ρ =−0.08 and ρ =−0.19 respectively).

T lymphocyte function was also defined by measuring IFN-γ production in cell culture supernatants after T cell stimulation. At D0, median natural log-transformed IFN-γ production was dramatically decreased after TCR stimulation compared to HV (2.62 [IQR: 2.34–3.55] *vs* 5.99 [IQR: 5.27–6.28] log IU/mL, *p* < 0.001, Fig. 3c). At D20, this production (5.47 [IQR: 5.12–5.63] log IU/mL) significantly increased compared with paired sampled obtained at D0 and no difference compared with normal values could be identified.

These results show that T cell function was markedly altered at ICU admission in critically ill COVID-19 patients in accordance with the development of a state of T exhaustion after severe SARS-Co-V2 infection. At D20, T cell function improved to return to normal values suggesting the COVID-19-induced T cell exhaustion might alleviate over time.

To note, we compared immune parameters between patients with or without concomitant bacterial infection at D0 and between patients with ICU-acquired infection before D20 vs patients with no secondary infections (Additional file 1: Table S4). At D0, IFN-γ production and T cell proliferations were not different between patients with or without concomitant bacterial infection. However, patients with simultaneous bacterial and viral infections at admission presented with increased PD-1 expressions on CD4 + and CD8 + T cells, higher PD-L1 expression on CD8 + T cells and CTLA4 expression on CD4 + T cells. At D20, no differences were observed for any immune parameter between patients with or without secondary bacterial infections. Thus the simultaneous presence of bacterial and viral infections may have amplified immune dysfunctions at ICU admission but these differences did not persist overtime in patients still in the ICU at D20. Because of the small number of patients in each group, these results need to be confirmed in a larger cohort of patients.

### Evaluation of rhIL-7 effect of T cell function ex vivo in COVID-19 patients

Finally, we investigated whether rhIL-7 could improve COVID-19-induced T cell exhaustion ex vivo. Based on previous results showing that major T cell alterations were identified at D0 in patients, we focused on samples obtained at ICU admission to evaluate rhIL-7 biological effect on T cell function.

**Fig. 4:**
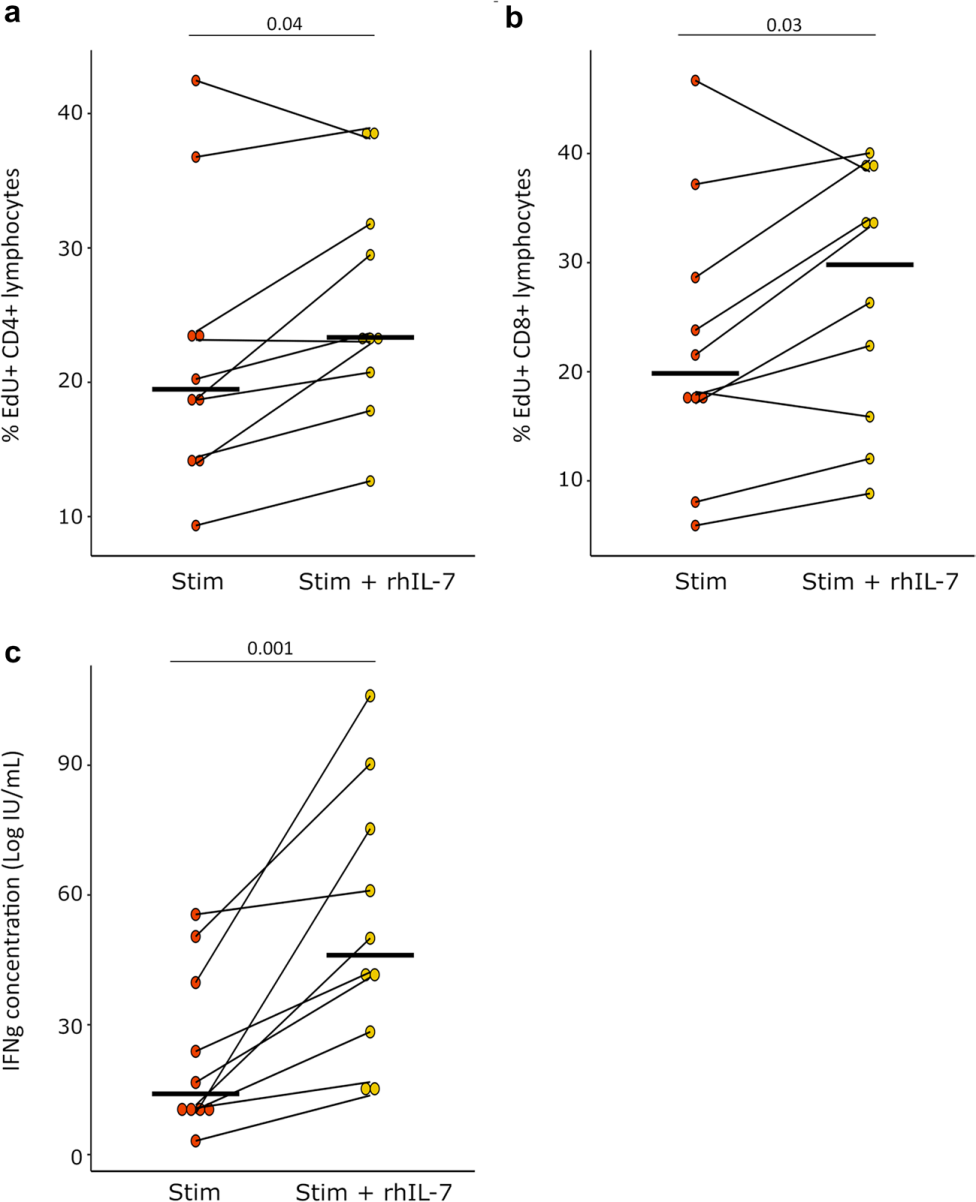
Lymphocyte proliferation and IFN-γ production after rhIL-7 and TCR stimulation in severe COVID-19 patients. PBMCs were collected in COVID-19 patients at D0 (*n* = 10). Cells were cultured for 3 days in the presence or not of anti-CD2/CD3/CD28 Ab-coated beads (TCR Stimulation, ratio of beads/cells = 2:1) and rhIL-7 (TCR Stimulatio* n* + rhIL-7,100 ng/ml). Cellular proliferation was assessed by monitoring EdU AF488 incorporation into cells and expressed as percentages of cells incorporating EdU. IFN-γ was measured in cell supernatants after proliferation. Results are presented as individual values and medians. **a** CD4 + T cell proliferation is presented. **b** CD8 + T cell proliferation is presented. **c** Natural log transformed IFN-γ production in culture supernatants is shown. Wilcoxon signed-rank test was used to compare results between groups. Only statistically significant differences are shown

We observed that rhIL-7 significantly increased both CD4 + and CD8 + T cell proliferations in critically ill COVID-19 patients. Median relative increase in proliferation was 21.8% [IQR: 11.3–33.7] and we observed a reproducible effect between patients (Fig. 4a and b). In addition, rhIL-7 showed a strong beneficial effect on IFN-γ production as it induced a 156% [IQR: 77.3–293.0] relative increase in IFN-γ production compared with cells stimulated without rhIL-7 (Fig. 4c).

Overall, these results showed that rhIL-7 presented with a strong beneficial effect on COVID-19-induced T cell exhaustion ex vivo in samples from COVID-19 patients.

## Discussion

Current results demonstrate that severe COVID-19 patients present with marked lymphopenia while remaining circulating lymphocytes show (i) altered transcriptomic profile with inhibition of T cell activation pathways, (ii) increased expressions of co-inhibitory receptors and (iii) altered functional responses ex vivo with decreased proliferation and IFN-γ production. These alterations, characteristic of T cell exhaustion, were more pronounced at ICU admission. rhIL-7 incubation ex vivo demonstrated a strong beneficial effect on COVID-19-induced T cell exhaustion ex vivo in samples from COVID-19 patients.

An increasing amount of data suggested that T-cell immunity plays an important role in eliminating SARS-CoV-2. Reports from hospitalized patients recovering without developing severe symptoms revealed emergence and rapid increase in activated CD38^+^HLA-DR^+^ T cells, especially CD8^+^ T cells, at days 7–9 after first presentation of symptoms [24]. This activation of T cells preceded the resolution of symptoms and might have prevented the evolution towards severe forms of the disease. Likewise, patients with mild symptoms who rapidly cleared the virus mounted robust T cell response, whereas only a weak and monospecific IFN-γ-secreting cell response was detected in deadly cases [25]. More importantly, viral clearance and reduced disease severity were associated with activation of both humoral and cellular anti-viral immune responses in COVID-19 patients as expansion of SARS-CoV-2 specific CD4 + T cells, CD8 + T cells and neutralizing antibodies have been shown to be beneficial in minimizing COVID-19 severity [26]. Overall, cell-mediated immune response seems to be of paramount importance in the fight against SARS-Co-V2 infection as a delayed specific T cell response is associated with disease severity [6].

Lymphopenia is a hallmark of severe COVID-19 and has been repeatedly associated with clinical severity and mortality [4, 27, 28]. In the present study, we confirmed the decreased lymphocyte count observed in severe COVID-19 patients affecting both CD4 + and CD8 + T cells. Similarly decreased lymphocyte number was observed in patients with bacterial sepsis [29, 30]. In septic patients also, alterations of lymphocyte count, phenotype or effector functions have been shown to be associated with deleterious outcomes [7].

In critically ill COVID-19 patients, we reported that remaining circulating T lymphocyte presented with multiple features of T cell exhaustion. This marked immunosuppressive profile was particularly present upon ICU admission. First, proliferative capacity of T cells from COVID-19 patients ex vivo was altered. Only few studies investigated this parameter so far in COVID-19. They similarly described altered T cell proliferation in severe patients [31, 32]. Several mechanisms could be involved in this reduced proliferative response: regulation through inhibitory checkpoint receptors, myeloid derived suppressor cell expansion [32, 33] or direct pathogenic effect of the virus SARS-CoV-2 itself because the expression of its receptor, angiotensin-converting enzyme 2, is increased on CD4 + T cells after ex vivo stimulation [34]. We also observed a dramatic decrease in IFN-γ production concomitant to this altered proliferative ability. This altered production of IFN-γ in severe cases of COVID-19 has been described before [11, 35, 36] but was not analyzed over time. We found that this production significantly improved at D20, suggesting that the impaired adaptive immune response may be involved early in COVID-19 immunopathology. Finally, increased expression of the co-inhibitory receptors PD-1, CTLA-4 or TIM-3 have been reported [35, 37, 38] and associated with disease severity in COVID-19 patients. We describe that this exhausted phenotype was probably related to the expression of multiple immune checkpoint inhibitors simultaneously and that this expression was sustained over time. These enhanced expressions could explain partly the lymphopenia observed in COVID-19 as increased PD-1 expression on CD4 + T cells was inversely correlated with decreased CD4 + T lymphocyte count. Similarly, in septic patients, apoptosis has been associated with increased PD-1 expression in CD4 + cells [39]. Overall, these results support the hypothesis that a dysregulated T lymphocyte response might contribute to the immunopathology of COVID-19 with the development of a state of T cell exhaustion in the most severe patients at ICU admission.

Only few studies focused on treatments to reverse COVID-19 related T cell exhaustion and rhIL-7 could be of interest in this setting [40]. IL-7 promotes lymphocyte survival and expansion but its expression declines with age-associated thymic atrophy [41]. Thus, rhIL-7 could help to overcome this pitfall by inducing increased thymopoiesis [42] and stimulating the generation of antigen-specific T cells [43]. In bacterial sepsis, preclinical studies showed that rhIL-7 treatment reduced mortality in murine models and improved cell functionality upon ex vivo activation of septic shock patients T lymphocytes [13–15]. A phase II clinical trial evaluating rhIL-7 in septic shock patients showed that IL-7 treatment restored T cell count in patients with severe lymphopenia in the absence of any severe side effects [44].

Herein, we report that ex vivo administration of rhIL-7 enhanced both CD4 + and CD8 + T cell proliferations and thus could participate in restoring the coordinated adaptive immune response required to control SARS-CoV-2 infection [26]. Furthermore, rhIL-7 improved IFN-γ lymphocyte production which is another aspect of COVID-19 lymphocyte dysfunction. Administration of rhIL-7 was efficacious at D0 so that our results support the rationale of using rhIL-7 early in the evolution of the disease. Similarly, two recent studies showed that ex vivo administration of rhIL-7 was associated with increased IFN-γ production of stimulated T cells in severe COVID-19 cases [11] and with restoration of IFN-γ production by T cells after treatment with dexamethasone [45]. Compassionate use in 12 critically ill patients with severe lymphopenia and in a case report allowed to restore lymphocyte count without exacerbating inflammation or pulmonary injury [17, 18]. One clinical trial investigating the effect of rhIL-7 in lymphopenic patients with COVID-19 infections is ongoing in Europe and in the USA (ILIAD-7, NCT04442178, NCT04407689, NCT04379076).

In the current cohort, most patients presented with alterations of T cell function at D0 but some patients may not display such immunosuppressed profile. Thus effect of rhIL-7 in these patients may not be as worthwhile. This implies careful selection of patients who would benefit from such immune-adjuvant therapy. Functional testing could be used to monitor patients’ actual adaptive immune response. However, despite representing the gold standard for functional testing, T cell proliferation assays may not be suitable to routinely monitor immune status in clinic because this test is time consuming, poorly standardized and it requires between 3 and 7 days of stimulation. Absolute lymphocyte count may not be an appropriate surrogate marker of T cell function as we did not observe any correlation between this parameter and lymphocyte proliferation in this study. Thus the appropriate stratification marker for rhIL-7 administration in critically ill COVID-19 patients remains to be determined.

Our study has some limitations that should be addressed. First, we focused on patients with the most severe forms of COVID-19 so that the T cell exhaustion profile observed may not be generalizable to all COVID-19 cases. Second, healthy donors and patients were not strictly age-matched. However we did not identify any significant correlation between increasing age of donors and PD1 expression on CD4 + T cells, PD-L1 expression on CD4 + T cells, PD-L1 expression on CD8 + T cells, CD4 + and CD8 + T cell proliferations. This argues against a strong effect of immunosenescence on immune parameters in this cohort. Third, we only assessed global T cell function and not SARS-CoV-2 specific T cell responses which may be affected in a different manner. Fourth, we did not assess whether parameters of IL-7 pathway might be altered before administering rhIL-7. Finally, the number of patients included in the cohort did not allow to define precisely which patients would respond the most to rhIL-7 therapy or if these lymphocyte alterations were associated with mortality. Both aspects now deserve to be confirmed in a larger cohort of critically ill COVID-19 patients.

## Conclusion

Our findings depict severe COVID-19 disease immunopathology as a defective T lymphocyte response related to a profound lymphopenia and exhausted T cells with decreased functionality. These alterations were present upon ICU admission and tended to improve over time. Importantly, this immunosuppressed phenotype was reversible ex vivo after rhIL-7 administration. Defining the appropriate timing for initiating such immune-adjuvant therapy in clinical setting and the pertinent markers for a careful selection of patients are now warranted to confirm the ex vivo results described so far.

## Supplementary Information

**Additional file 1.** Additional tables.

## Data Availability

The datasets analyzed during the current study are available from the corresponding author on reasonable request.
