# Supplementary material for: Recombinant human interleukin-7 reverses T cell exhaustion ex vivo in critically ill COVID-19 patients
Source: Ann Intensive Care. 2022 Mar 5;12:21. doi: 10.1186/s13613-022-00982-1 (PMC8896969; doi:10.1186/s13613-022-00982-1)
Supplement: Supplementary file 1 — Additional file 1. Additional tables. [file 13613_2022_982_MOESM1_ESM.pdf]

SUPPLEMENTAL INFORMATION

Recombinant human interleukin-7 reverses T cell exhaustion *ex vivo* in critically ill COVID-19 patients

Frank Bidar[1,2,3], Sarah Hamada[3], Morgane Gossez[3,4], Remy Coudereau[1,3], Jonathan Lopez[5], Marie-Angelique Cazalis[1], Karen Brengel-Pesce[1], Marine Mommert[1], Marielle Buisson[6], Filippo Conti[1], Thomas Rimmelé[1,2], Anne-Claire Lukaszewicz[1,2], Laurent Argaud[7], Martin Cour[7], Guillaume Monneret[1,3], Fabienne Venet[1,4] for the RICO study group

SUPPLEMENTAL TABLES

Table S1. Significantly up- and down-regulated genes in COVID-19 patients at Day 0 compared with healthy volunteers.

RNA extracted from PBMCs of COVID-19 patients at day 0 (n = 10) and healthy volunteers (n= 10) was analyzed through NanoString technology using the nCounter® PanCancer Immune Profiling Panel. Differentially expressed genes were determined based on Log2 Fold Change ≤ -2 or ≥ +2 and –Log10 P value ≥ 1.3.

Gene	Log2 Fold Change	- Log10 P value
FCER1A	-4,030136106	2,7722232
MAGEC1	-2,990849995	1,68029283
MAGEB2	-2,689119163	1,40684639
IL13RA2	-2,670742456	1,48848743
CT45A1	-2,670639343	1,87594691
MAGEA12	-2,655671021	1,36052205
MAGEC2	-2,548592408	1,60612648
SSX1	-2,493230658	1,59360809
SSX4	-2,363970242	1,49859545
LRRN3	-2,173542243	6,49294414
CLEC4C	-2,153950429	1,63692438
MAGEA3	-2,09153476	1,31786124
MX1	2,01333371	2,84970316
IFI35	2,024585647	6,17767815
KIR Inhibiting Subgroup 2	2,174014839	2,47451132
MERTK	2,394149577	2,15285809
ITGA1	2,494918032	1,72412802

LAG3	2,501075871	3,63041581
S100A8	2,558030351	2,08421554
KIR Activating Subgroup 1	2,637419648	1,47096808
IL10	2,785818168	5,10873957
TNFRSF13B	2,847791006	1,4626652
ITGA2B	3,071505841	1,76670372
CD9	3,358146703	1,37578052
C1R	3,378535085	2,85497418
IL1R2	3,650076939	2,17992363
ITGB3	3,717991109	1,44031251
CLEC5A	4,558801643	1,44938719
IFI27	5,398161312	2,33778032

Table S2. Significantly up- and down-regulated genes in COVID-19 patients at Day 20 compared with healthy volunteers.

RNA extracted from PBMCs of COVID-19 patients at day 20 (n = 10) and healthy volunteers (n= 10) was analyzed through NanoString technology using the nCounter® PanCancer Immune Profiling Panel. Differentially expressed genes were determined based on Log2 Fold Change ≤ -2 or ≥ +2 and –Log10 P value ≥ 1.3.

Genes	Log2 Fold Change	- Log10 Pvalue
MAGEA12	-3,73566265	1,53609544
MAGEC1	-3,564373226	1,77781345
IL13RA2	-3,563686769	1,65188203
MAGEB2	-3,352594519	1,52725952
CT45A1	-3,161525597	1,99292891
SSX4	-3,123372985	1,6883063
MAGEC2	-3,065943891	1,73224936
SSX1	-3,011822394	1,72352808
SPANXB1	-2,802842236	1,37306062
MAGEA3	-2,780736615	1,51785959
MAGEA1	-2,723373482	1,33353839
IL1RAPL2	-2,697516753	1,53077792
SELE	-2,468438816	1,65070709
CD1E	-2,455661163	1,4251441
CCL22	-2,454938616	1,50825996
C3	-2,42635283	1,41183176
C8B	-2,366968247	1,46491332
IL2	-2,351749839	1,67094432
IFNA8	-2,34265308	1,57008462
CCL16	-2,313595764	1,5315635
CX3CL1	-2,30863901	1,33968793
IL22RA1	-2,305036824	1,48951473
KIR3DL3	-2,289798317	1,35678979
MST1R	-2,28624901	1,47874847
LRRN3	-2,282762787	6,58817834
C8A	-2,265718073	1,54590641
C1S	-2,249822745	1,37611987
LBP	-2,239973536	1,55460694
CCL18	-2,231675818	1,56127954
IL12B	-2,206348593	1,45024769
CD207	-2,195184025	1,55451134
CD209	-2,187279839	1,46136254
CCL13	-2,186382688	1,51385032
CCL27	-2,153611966	1,33036022

BID	-2,150230303	1,45118973
RAG1	-2,14408087	1,42003853
SEMG1	-2,111716286	1,58265613
DDX43	-2,103242343	1,61920258
IL21	-2,082930504	1,46994919
LILRA4	-2,075710439	3,39552409
IFNB1	-2,056977301	1,49837503
CCL8	-2,037012026	1,58852413
PRM1	-2,023483901	1,50882999
SPO11	-2,022892719	1,39795826
CCL1	-2,005634	1,3862372
A2M	-2,000680886	1,35288527
SBNO2	2,176608318	3,98902792
S100A12	2,346749821	1,5745425
LAG3	2,348068623	1,68870554
S100A8	2,351324395	1,73769687
PPBP	2,356841691	2,49152915
MERTK	2,415646828	2,12143447
ITGA1	2,737598798	2,46431782
ITGA2B	2,836680704	3,19584199
CD9	3,120203313	2,43607764
SERPINB2	3,137942192	1,33439115
ITGB3	3,269622192	3,15632818
C1R	3,29181307	2,67281122

Table S3. Canonical pathways differentially expressed between COVID-19 patients at Day 0 or Day 20 and healthy volunteers

RNA extracted from PBMCs of COVID-19 patients at day 0 and day 20 (n = 10) and healthy volunteers (HV, n= 10) were analyzed through NanoString technology using the nCounter® PanCancer Immune Profiling Panel. List of differentially expressed genes were determined after normalization based on Log2 Fold Change ≤ -2 or ≥ +2 and –Log10 P value ≥ 1.3. Significantly enriched canonical pathways were subsequently identified through Ingenuity Pathways Analysis. Corresponding Z-score and -log10 p values are reported.

Pathways	Z Score (Day 0)	Log10 P value (Day 0)	Z Score (Day 20)	Log10 P value (Day 20)
IL-23 Signaling Pathway	-0,447	5,63	-0,707	11,00
IL-17 Signaling	-0,905	8,86	-1,807	11,90
IL-15 Signaling	2,236	4,52	0,447	5,03
IL-15 Production	1,342	3,56	0,302	10,70
T Cell Receptor Signaling	0,447	0,84	-0,302	3,39
Th17 Activation Pathway	-0,277	6,59	-0,277	6,58
Th2 Pathway	-1,667	11,7	-1,508	11,40
Th1 Pathway	-0,447	6,95	-1,667	9,44
Activation of IRF by Cytosolic Pattern Recognition Receptors	0,378	7,66	-2,333	11,00
Acute Phase Response Signaling	0,447	4,56	-0,333	9,69
B Cell Receptor Signaling	2	1,85	1	3,56
Breast Cancer Regulation by Stathmin1	-0,632	3,33	-0,816	0,76
Colorectal Cancer Metastasis Signaling	1,89	3,52	-0,333	5,01
Crosstalk between Dendritic Cells and Natural Killer Cells	1,633	6,61	-2,111	15,20
Dendritic Cell Maturation	-0,333	2,87	-1,886	8,46
Erythropoietin Signaling Pathway	1,897	7,84	2,111	8,69
Estrogen Receptor Signaling	2	0,91	1,342	1,35
Hepatic Fibrosis Signaling Pathway	0,333	6,11	0,277	9,59
HER-2 Signaling in Breast Cancer	1,134	4,41	1,508	7,29
IL-8 Signaling	2,236	3,29	1,667	5,95
ILK Signaling	1	1,84	0,447	2,77
iNOS Signaling	2	5,45	2	9,06
Integrin Signaling	2,236	2,48	2,121	4,18
Interferon Signaling	1,89	9,54	-0,816	7,03
LPS/IL-1 Mediated Inhibition of RXR Function	-0,447	2,89	-1	1,63
LXR/RXR Activation	-0,333	7,99	-0,333	8,00
MSP-RON Signaling In Macrophages Pathway	1,134	5,82	0,707	7,15
Natural Killer Cell Signaling	1,89	4,32	0,832	10,30
Neuroinflammation Signaling Pathway	1,508	7,33	-1,606	14,60

NF-kB Signaling	0,632	3,61	-0,302	3,14
p38 MAPK Signaling	-0,447	4,72	-0,816	6,08
PPARa/RXRa Activation	-0,447	2,63	0	2,07
Production of Nitric Oxide and Reactive Oxygen Species in Macrophages	2	1,87	0,378	5,34
Regulation Of The Epithelial Mesenchymal Transition By Growth Factors Pathway	1,134	4,48	0,378	4,52
Role of Hypercytokinemia/hyperchemokinemia in the Pathogenesis of Influenza	1,265	11	-3,207	15,50
Role of NFAT in Regulation of the Immune Response	1	0,89	0,707	1,98
Role of Pattern Recognition Receptors in Recognition of Bacteria and Viruses	1,89	15,2	-2,111	18,50
Role of RIG1-like Receptors in Antiviral Innate Immunity	0,447	5,63	-1,89	9,36
Semaphorin Neuronal Repulsive Signaling Pathway	-2	3,12	-1,633	5,23
STAT3 Pathway	1	5,46	-0,816	7,80
Systemic Lupus Erythematosus In B Cell Signaling Pathway	0,775	11,4	-3,128	19,60
Systemic Lupus Erythematosus In T Cell Signaling Pathway	0,302	3,8	-0,333	1,76
Toll-like Receptor Signaling	0,447	9,94	-1,134	16,10
TREM1 Signaling	0,447	4,49	-0,378	7,48
Tumor Microenvironment Pathway	0,816	3,6	1,134	5,51

Table S4. Immune parameters in patients with or without concomitant bacterial infections at D0 and D20. Results were compared between patients with or without concomitant bacterial infection at D0 (n = 9 co-infected patients vs 11 with only SARS-CoV-2 infection) and between patients with ICU-acquired infection before D20 (n = 15) vs patients with no secondary infections (n = 5). Comparisons between groups were made using non parametric Mann-Whitney U tests.

**At D0**	**bacterial co-infection: NO**	**bacterial co-infection: YES**	**p-value**
IFN-γ production (IU/mL)	14.02 [10.91 – 33.98]	17.10 [8.55 – 31.76]	0.831
T cell proliferation (% Edu+ CD3+ T cells)	19.68 [18.47 – 20.72]	14.42 [11.51 – 22.95]	0.286
PD-1 expression on CD4+ T cells (% positive cells)	12.03 [8.13 – 14.61]	28.06 [22.98 – 28.11]	**0.012**
PD-1 expression on CD8+ T cells (% positive cells)	12.21 [11.43 – 17.64]	25.22 [23.22 – 36.23]	**0.042**
PD-L1 expression on CD4+ T cells (% positive cells)	1.4 [0.24 – 3.01]	3.59 [2.00 – 6.42]	0.167
PD-L1 expression on CD8+ T cells (% positive cells)	2.18 [1.89 – 3.17]	12.57 [6.96 – 13.65]	**0.019**
CTLA4 expression on CD4+ T cells (% positive cells)	0.16 [0.10 – 1.49]	3.28 [2.24 – 4.57]	**0.019**
CTLA4 expression on CD8+ T cells (% positive cells)	0.53 [0.30 – 2.60]	3.06 [2.70 – 4.68]	0.088
TIM3 expression on CD4+ T cells (% positive cells)	1.13 [0.52 – 4.19]	3.96 [3.90 – 4.91]	0.123
TIM3 expression on CD8+ T cells (% positive cells)	4.24 [1.77 – 7.28]	8.38 [7.30 – 10.40]	0.167
**At D20**	**bacterial co-infection: NO**	**bacterial co-infection: YES**	**p-value**
IFN-γ production (IU/mL)	399.50 [306.88 – 913.00]	206.00 [167.38 – 487.62]	0.439
T cell proliferation (% Edu+ CD3+ T cells)	18.11 [15.36 – 19.39]	26.70 [15.93 – 40.78]	0.439
PD-1 expression on CD4+ T cells (% positive cells)	10.14 [7.01 – 16.25]	23.74 [17.63 – 26.36]	0.079
PD-1 expression on CD8+ T cells (% positive cells)	34.26 [21.27 – 34.42]	26.62 [18.63 – 50.98]	0.518
PD-L1 expression on CD4+ T cells (% positive cells)	0.03 [0.03 – 2.39]	1.54 1.37 – 2.58]	0.405
PD-L1 expression on CD8+ T cells (% positive cells)	1.04 [0.73 – 8.61]	4.42 [3.90 – 8.73]	0.309
CTLA4 expression on CD4+ T cells (% positive cells)	0.09 [0.07 – 1.39]	1.97 [1.52 – 3.00]	0.166
CTLA4 expression on CD8+ T cells (% positive cells)	0.28 [0.23 – 2.21]	2.97 [2.03 – 3.21]	0.229
TIM3 expression on CD4+ T cells (% positive cells)	0.40 [0.30 – 6.72]	3.39 [2.38 – 6.89]	0.518
TIM3 expression on CD8+ T cells	1.91	7.27	0.518

(% positive cells)	[1.26 – 14.87]	[5.25 – 10.62]	
